# Three-Dimensionally Printed Microstructured Hydrophobic Surfaces: Morphology and Wettability

**DOI:** 10.3390/polym17192570

**Published:** 2025-09-23

**Authors:** Loredana Tammaro, Sergio Galvagno, Giuseppe Pandolfi, Fausta Loffredo, Fulvia Villani, Anna De Girolamo Del Mauro, Pierpaolo Iovane, Sabrina Portofino, Paolo Tassini, Carmela Borriello

**Affiliations:** 1 Laboratory Smart Components and Systems for Sustainable Manufacturing, Department for Sustainability, Division Technologies and Materials for Sustainable Manufacturing Industry, ENEA Research Center, 80055 Portici, Italy; sergio.galvagno@enea.it (S.G.); giuseppe.pandolfi@enea.it (G.P.); fausta.loffredo@enea.it (F.L.); fulvia.villani@enea.it (F.V.); pierpaolo.iovane@enea.it (P.I.); sabrina.portofino@enea.it (S.P.); paolo.tassini@enea.it (P.T.); carmela.borriello@enea.it (C.B.); 2 Innovative Devices Laboratory, Energy Technologies and Renewable Sources Department, Solar Photovoltaics Division, ENEA Research Center, 80055 Portici, Italy; anna.degirolamo@enea.it

**Keywords:** fused filament fabrication (FFF), polylactic acid (PLA), hydrophobicity, surface treatment

## Abstract

This work presents the design and fabrication of microstructured hydrophobic surfaces via fused filament fabrication (FFF) 3D printing with polylactic acid (PLA). Three geometric patterns—triangular-based prisms (TG), truncated pyramids (TP), and truncated ellipsoidal cones (CET)—were developed to modify the surface wettability. Morphological analysis revealed that the printer resolution limits the accurate reproduction of sharp CAD-defined features. Despite this, TG structures exhibited superhydrophobic behavior evaluated through static water contact angles (WCAs), reaching up to 164° along the structured direction and so representing a 100% increase relative to flat PLA surfaces (WCA = 82°). To improve print fidelity, TP and CET geometries with enlarged features were introduced, resulting in contact angles up to 128°, corresponding to a 56% increase in hydrophobicity. The truncated shapes enable the fabrication of the smallest features achievable via the FFF technique, while maintaining good resolution and obtaining higher contact angles. In addition, surface functionalization with fluoropolymer-coated SiO_2_ nanoparticles, confirmed by SEM and Raman spectroscopy, led to a further slight enhancement in wettability up to 18% on the structured surfaces. These findings highlight the potential of FFF-based microstructuring, combined with surface treatments, for tailoring the wetting properties of 3D-printed polymeric parts with promising applications in self-cleaning, de-icing, and anti-wetting surfaces.

## 1. Introduction

Hydrophobic and superhydrophobic surfaces are gaining attention in a variety of high-performance and industrial applications due to their ability to repel water, resist contamination, and enhance the durability of exposed components. Inspired by natural phenomena such as the lotus leaf effect and insect wings, these surfaces exhibit minimal water adhesion, enabling droplets to roll off easily and carry away contaminants, and are characterized by low surface energy and micro/nanostructured topographies, which together result in high water contact angles and low adhesion forces. Therefore, they are increasingly being integrated into sectors that demand long-term environmental resistance and mechanical robustness, including outdoor electronics housing, biomedical devices, packaging, and automotive components [1,2,3,4].

The functional benefits of these surfaces extend well beyond water repellency. Their self-cleaning, anti-fouling, anti-icing, and corrosion-resistant properties make them ideal for use in environments where materials are exposed to moisture, pollutants, or biological agents. For instance, in outdoor electronics, superhydrophobic coatings protect sensitive components from humidity and water ingress, thereby improving reliability and lifespan.

In biomedical devices, these surfaces can reduce bacterial adhesion and biofilm formation, contributing to improved hygiene and reduced infection risks. In packaging, especially for food and pharmaceuticals, they help to maintain product integrity by preventing moisture accumulation and contamination. In the automotive and aerospace sectors, superhydrophobic treatments are used to reduce drag, prevent icing on surfaces, and protect against environmental degradation. In microfluidics and lab-on-a-chip technologies, superhydrophobicity plays a crucial role in controlling fluid motion and minimizing sample loss. Moreover, hydrophobic surfaces find applications such as self-cleaning materials (e.g., windows, textiles, solar panels).

The fabrication of such surfaces typically involves two key elements: surface roughness at micro- and nanoscales and low-surface-energy materials. The main fabrication strategies imply (1) engineering hierarchical surface textures through techniques such as chemical etching, plasma treatment, lithography, sol–gel processes, and laser texturing [5], and (2) applying nanoparticle coatings composed of low-surface-energy materials like fluoropolymers or silicones [6]. These approaches can be used independently or in combination to achieve the desired wetting behavior.

More recently, among the various methods employed to achieve hydrophobic surfaces, additive manufacturing, particularly fused filament fabrication (FFF), has emerged as a versatile and economic approach for designing and tailoring surface topographies with high precision and reproducibility. The process operates by melting and depositing thermoplastic material layer by layer in accordance with a predefined Computer-Aided Design model (CAD model). By adjusting printing parameters such as layer height, nozzle temperature, and infill pattern, it is possible to tailor the surface roughness and energy, both of which are critical factors in enhancing hydrophobicity.

Moreover, the flexibility of FFF in terms of material selection, including polymers that can be chemically modified or blended with hydrophobic additives, further broadens its applicability in this field. Materials such as polylactic acid (PLA) are commonly employed in FFF due to its favorable thermal and rheological properties. PLA is a biodegradable aliphatic polyester synthesized from renewable resources such as corn starch or sugarcane [7,8]. Its promising chemical–physical properties, including high mechanical strength, low toxicity, and ease of processing, have made it one of the most widely used thermoplastics in additive manufacturing, particularly in FFF [9,10]. Also, it needs less energy and temperature to process prototypes and functional parts with good quality. However, PLA’s intrinsic hydrophilicity limits its performance in applications where moisture resistance and surface durability are critical.

To overcome these limitations, recent research has focused on engineering PLA surfaces to exhibit hydrophobic or even superhydrophobic behavior. This has been achieved through two main strategies: (i) optimization of FFF printing parameters to induce micro- and nanoscale surface roughness, and (ii) post-processing treatments such as chemical coatings or plasma treatments that reduce surface energy [11,12,13,14,15,16,17,18,19]. For instance, Yang et al. fabricated a three-dimensional grid-like solid model featuring holes measuring 1.5 mm × 1.5 mm, spaced 1.5 mm apart. They employed four distinct infill patterns—rectilinear, grid, wiggle, and honeycomb—to fabricate the samples. They verified that both the layer thickness and the filling method significantly affected the surface roughness of the 3D-printed specimens, which in turn influenced their hydrophobic properties. The highest contact angle observed was 103° [12].

Borriello et al. demonstrated that micropatterned hydrophobic 3D surfaces, fabricated via FFF using PP, PP/carbon fiber, and PLA/carbon nanotube composite filaments, significantly increased water contact angle—up to 50% compared to flat surfaces—by varying pillar dimensions and spacing. This enhancement was achieved solely through microstructuring, without chemical treatments or nanoparticles [13]. Amin et al. reported different sizes of pyramid shapes made using PLA-based materials via the FFF 3D-printing method. The surface of the 3D-printed samples was then subjected to chemical etching using hydrogen fluoride to further increase the roughness of the samples. The maximum WCA of 142° was obtained for an etched sample having square pyramid (edge of base of 1.5 mm) surface morphology and altitude of 1.5 mm [17]. Furthermore, Bañón-García et al. identified the highest water contact angle (WCA) for a 3D-designed pyramid geometry featuring a width of 1.0 mm, a low height of 0.15 mm, and no gap between structures. This configuration exhibited a WCA of 81° in the direction perpendicular to the drop deposition [19]. Manabe et al. developed a biomimetic, armadillo-inspired 3D-printed structure with a highly durable superhydrophobic surface by combining a hexagonal structure with varying areas, hydrophobic nanoparticles, and a photocurable resin [14].

Moreover, the incorporation of functional additives such as fluorinated siloxanes or organosilicon compounds into PLA matrices has shown promising results in enhancing hydrophobicity and anti-icing properties. For instance, Konieczna et al. reported that 3D-printed PLA materials through the incorporation of fluorinated-siloxane derivatives exhibited water contact angles that increased up to 39%, while maintaining or even improving mechanical performance [18]. Based on the literature review, the most effective microstructured geometries for enhancing water repellency are pillar-based patterns, particularly cylindrical, polygonal, and conical pillars arranged in regular lattices [4,20]. These geometries promote stable air entrapment and minimize droplet adhesion, allowing water to bead up and roll off easily. The regularity of the lattice ensures uniformity in surface energy distribution, which contributes to consistent hydrophobic performance across the surface.

In this work, patterns of pillars with three different geometries—triangular-based prism, truncated pyramid, and truncated ellipsoidal cone—were investigated by varying their dimensions and spacing between patterns to achieve a rougher PLA surface aiming to improve the hydrophobicity of 3D substrates. The implementation of variations to geometries commonly used in the literature enabled the fabrication of smaller features using the FFF technique.

The morphology of the printed structures was analyzed using optical microscopy to assess the compliance of the 3D-printing process and to evaluate the accuracy of the fabricated geometries by comparing them with the corresponding CAD models. The wettability of the structured samples was determined through static water contact angle (WCA) measurements. Furthermore, the influence of a hydrophobic coating composed of fluoropolymer-functionalized SiO_2_ nanoparticles on surface wettability was tested on the best-performing geometries. The resulting modifications in wettability were then evaluated.

## 2. Materials and Methods

### 2.1. Materials

A commercial PLA-based filament (Galaxy Silver; diameter 1.75 mm ± 0.05 mm) was purchased from Prusa Research (Prague, Czech Republic). Prior to processing, it was dried in an oven at 80 °C for 4 h to remove moisture.

Tetraethyl orthosilicate (TEOS) and trichloro(octadecyl)silane (OTDS) were obtained from Sigma-Aldrich (Darmstadt, Germany). The fluoropolymer Fluorolink S10 was purchased from Solvay, Kanto Chemical (Tokyo, Japan). All reagents and materials were used as received without further purification.

### 2.2. Fabrication of 3D Samples

For the fabrication of 3D specimens, a commercially available FFF 3D printer Prusa i3 MK3S was used. Various patterns showing different microstructured geometries, with varying shape, heights, and interstructural spacing distance were designed using SolidWorks 2022 software. The designs were exported into Standard Tessellation Language (STL) and processed by the last version of Prusaslicer software (version 2.9.2) to obtain the instruction files for the 3D printer (gcode). Flat 3D samples measuring 2.4 × 1 cm^2^ and patterned samples of 2.4 × 2.4 cm^2^ were printed. All dimensions of generated geometries are provided in the Supplementary Materials.

Various combinations of nozzle temperatures (ranging from 195 °C to 220 °C), bed temperatures (between 55 °C and 60 °C), and printing speeds (from 15 to 40 mm/s) were systematically tested, resulting in the production of numerous samples. The optimal set of parameters was selected to ensure the highest reproducibility and finest microstructural resolution. The key printing parameters are summarized in Table 1.

### 2.3. Synthesis of Silica Nanoparticles (SiO_2_ NPs)

Silica nanoparticles were synthesized according to a revised procedure reported in the literature [21] starting from tetraethyl orthosilicate (TEOS) and subsequently treating with trichloro(octadecyl)silane (OTDS) and the fluoropolymer Fluorolink S10. Fluoropolymer-coated nanoparticles SiO_2__S10 isopropanol suspension was obtained and deposited on 3D-printed sample surfaces by dip coating. The nanoparticles dispersion remained stable for up to 24 h; after this period, a small amount of sediment became visible at the bottom of the container.

### 2.4. Characterization Methods

The sample morphology was studied through microscopy analyses carried out by an AXIO Scope A.1 microscope (Zeiss, Jena, Germany) in reflection mode by using a 5× magnification. The measurements of the pattern dimensions and the spacing distance among patterns in x and y directions were measured by Zen Core 2.7 software, from the optical micrographs performed in different regions of the samples. The reported average values were calculated from at least 15 measurements obtained at different points.

To evaluate the pillar height (h), which could not be directly measured using the AXIO Scope A.1 optical microscope, the surface profile of the 3D-printed PLA specimens was characterized by a VHX-X1 series Keyence Digital Microscope equipped with a 4K high-resolution VHX-7100 color camera controlled through H6M software (version 3.3.25.641).

The surface wettability was evaluated by contact angle measurements of deionized water (WCA) by sessile drop method using an OCA 20 (Dataphysics, Filderstadt, Germany) goniometer, and data were collected with SCA 202 software (version 3.4.3 build 76). Equilibrium (static) contact angles were measured for 1 μL droplet volumes. Measurements were made in 10 different locations for each condition, and the average value was reported with the standard deviation.

Morphological analyses of the samples coated with SiO_2__S10 nanoparticles were carried out using a scanning electron microscope (SEM, Carl ZEISS LEO 1530 SMT GmbH, Oberkochen, Germany) equipped with a filament of tungsten at 4 kV and working under high-vacuum conditions.

The Raman spectroscopy was performed by a Renishaw InVia Reflex (Renishaw, Torino, Italy) spectrometer by using a laser with wavelength of 514.5 nm (laser power of 50%) and a 50× magnification objective. The investigated wavelength range was 300–1400 cm^−1^. Each measurement consisted of 20 consecutive accumulations with an exposure time of 20 s. A total of 8 measurements were performed for each sample.

## 3. Results and Discussion

Through 3D-printing technology, it is possible to create structures that enhance the hydrophobic character of materials. To further increase this property, it is necessary to improve both the geometry and the quality of the printed parts and, if required, apply surface treatments. In this frame, the main goal of our work was to evaluate the feasibility of creating surface structuring using fused filament fabrication (FFF) 3D-printing technology.

### 3.1. FFF 3D-Printed Geometries

New geometries were designed and analyzed using PLA-based filaments. Pointed structures (TG, triangular-based prism) and two types of flat-based structures (TP, truncated pyramid, and CET, truncated ellipsoidal cone) were designed and printed, leaving concave cavities between each structure. The dimensions of the structures and their spacing in two directions (x and y) were also varied. For comparison, a flat surface (F) was printed using the same printing parameters. Numerous samples of the three selected geometries were fabricated (reported in the Supplementary Materials) to find the best compromise between FFF 3D-printing resolution and wettability properties. Table 2 reports the geometric characteristics and CAD design of the samples that yielded the best results.

### 3.2. Characterization

**Figure 1:**
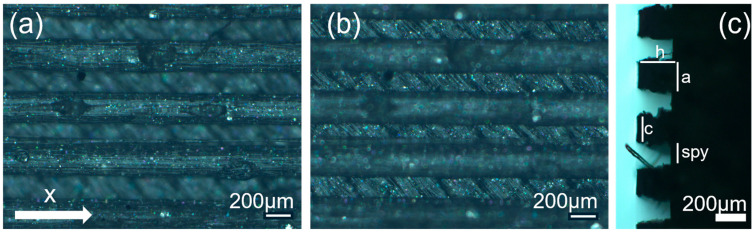
Optical microscopy images of the top (**a**), bottom (**b**), and side profile (**c**) of the TG sample.

Figure 1 presents the morphological characterization of the TG pattern performed using an optical microscope. It can be observed that the printed TG sample does not exhibit a triangular prism profile as intended but rather a trapezoidal one, with a highly irregular surface. As reported in Table 3, shorter side of the lower base (a) measures approximately 260 µm, while the shorter side of the upper base (c) is about 200 µm. The distance (spy) between the bases is not zero, as originally projected in the CAD design of the TG structure, but approximately 130 µm.

**Figure 2:**
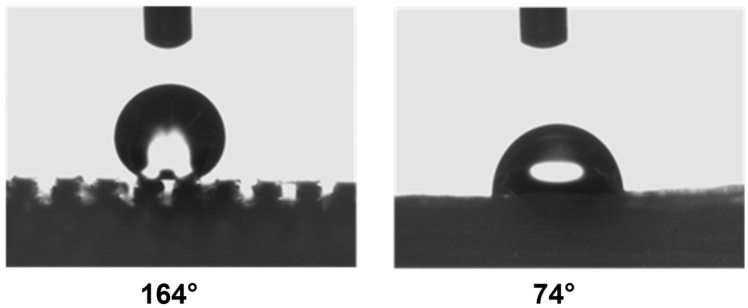
Wettability measurements of the TG sample along the y-direction (**left**) and x-direction (**right**).

**Figure 3:**
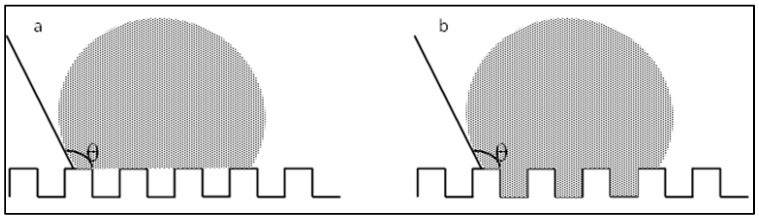
Model Cassie–Baxter (**a**) and Wenzel (**b**).

This evidence highlights that, in the adopted experimental conditions, the resolution of the printer used for the fabrication of the TG pattern is not sufficient to reproduce the designed pointed structures accurately, resulting in discrepancies in both geometric shape and dimensions. However, the TG sample exhibits superhydrophobic characteristics along the structured direction (y), with a contact angle of about 164° (Figure 2) and the typical behavior of a Cassie–Baxter model [22] (Figure 3), where the water droplet is suspended between two consecutive structures, supported by an air cushion.

The trapezoidal geometry (truncated pyramid) proved to be effective in achieving superhydrophobicity, both due to its compact dimensions and its specific shape. The sloped sidewalls of the truncated pyramid reduce the solid–liquid contact area and facilitate air entrapment beneath the droplet, promoting a Cassie–Baxter wetting regime. Furthermore, optical microscopy revealed a pronounced staircase effect, which probably enhances the retention of air pockets between layers, further contributing to the hydrophobic behavior. Naturally, along the x-direction—where the sample appears as a continuous line—the contact angle is almost that of an unstructured printed sample (74°).

Given the limitations of the printer used and the results obtained with the TG geometry, a new structure was designed with a shape like the resulting TG sample—namely, a trapezoidal profile (TP)—but with larger dimensions, more easily achievable by the printer.

Table 2 presents the geometric and dimensional characteristics of the TP samples (TP0, TP1). In TP0 the dimensions b and d are equal to the specimen length, while TP1 sample was fabricated preserving the same geometric and dimensional features of TP0 along the y-axis but introducing a similar structure along the x-axis. In TP1, as shown in the top view of the CAD model of Table 2, the truncated pyramids in adjacent rows along the x-direction are arranged in a staggered configuration. This design choice aims to partially compensate for the larger dimensions of b and d along the x-axis compared to c and d along the y-axis, while minimizing the possible introduction of water drops in both directions.

A comparison between fabricated pyramid geometries and the literature data reveals that the truncated pyramid structure (sample TP0) exhibits a significantly higher water contact angle (WCA), reaching up to 151°. In contrast, the best-performing square-based pyramid geometry reported in the literature [17]—despite being designed with a pointed tip—achieved a WCA approximately 20° lower than TP0, with structural dimensions of height: 1.5 mm, width: 1.5 mm, and gap: 0.

**Figure 4:**
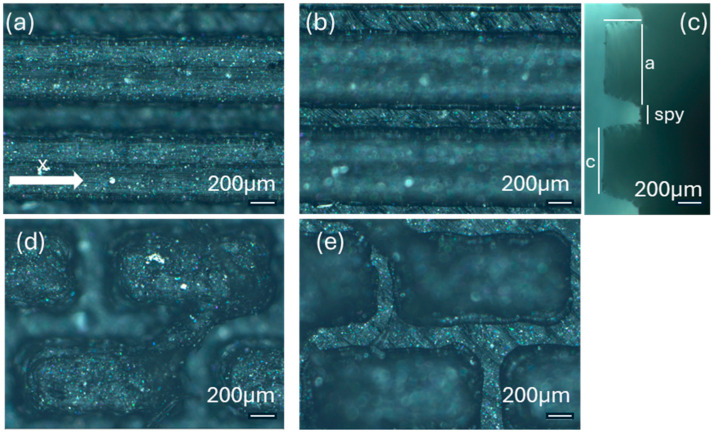
Optical microscopy images: (**a**) top, (**b**) bottom, and (**c**) side profile of TP0 sample; (**d**) top and (**e**) bottom of TP1 sample captured by AXIO Scope A.1 microscope.

**Figure 5:**
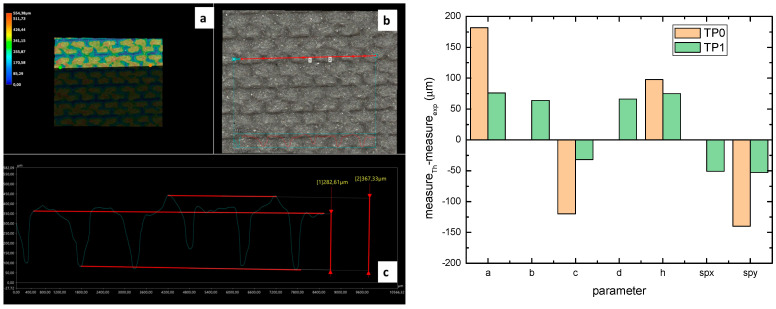
Left: (**a**) optical and digital composition of 3D images, (**b**) optical microscopy image of the top surface, (**c**) profile analysis of sample TP1 (scanned along the red line of (**b**))**.** Right: deviations between theoretical (th) and experimental (exp) dimensions of TP0 and TP1 structures.

Figure 4 reports the optical images of the structures obtained for the samples TP0 and TP1. In the lateral view image of TP0 (Figure 4c), a trapezoidal shape is confirmed; moreover, along with occasional filament bridging between adjacent structures. The bottom surface displayed the characteristic striated appearance commonly observed in structures fabricated using FFF technology [23,24]. Due to the staggered arrangement of the TP1 structures, a clear side profile of the sample could not be obtained using the AXIO Scope A.1 microscope. Instead, the evaluation of the side profile was carried out using the VHX-X1 Keyence digital microscope, as shown in Figure 5.

Sometimes, filamentary bridges can also be observed between the patterned structures. These unwanted lightweight connections are formed by the transport of the molten polymer during nozzle movements; known as stringing, they are typical of the FFF technique and, due to the very small dimensions of the patterns, are difficult to compensate for by adjusting the retraction parameters.

Nevertheless, the stringing effect seems to be relatively homogeneously distributed throughout the sample, averaging out its influence on the surface properties.

A comparison of the printing accuracy between TP0 and TP1 was carried out by measuring seven parameters of the structures—a, b, c, d, h, spx, and spy—as detailed in Table 4. The dimensional deviations (in µm) between theoretical and experimental average values for both types of TP structures are illustrated in the bar graph in Figure 5, with TP0 represented by orange bars and TP1 by green bars. For TP1 sample, characterized by a staggered sequence of structures, the h dimension was evaluated through digital optical microscope.

Similar types of statistical errors are observed for the two types of printed samples concerning dimensional accuracy. Indeed, referring to the histograms in Figure 5, for both samples positive deviations were measured for most structural parameters except for c and the spacing between the structures, which were larger than expected. This indicates that the experimental values of a, b, d, and h are smaller than theoretical ones, while c, spx, and spy are larger in both TP0 and TP1 samples. The TP1 sample, which features fully constrained dimensions, showed a closer match to the CAD model compared to TP0, as reported in Table 4 and Figure 5. Indeed, TP1 shows lower or comparable deviations for all the measured parameters, particularly for a, c, spx, and spy, which showed minimal deviations. The height h of TP1, measured by optical digital microscopy, was 325 ± 42 µm, whereas the expected value was 400 µm. It should be noted that the h measurement was averaged over a small area, which may affect the accuracy of the result.

In contrast, TP0 displayed significant discrepancies from the nominal values: the a dimension was reduced (618 ± 54 µm vs. 800 µm), the c dimension was enlarged (520 ± 27 µm vs. 400 µm), the height h was lower than expected (302 ± 3 µm vs. 400 µm); the spacing between the patterns, designed to be zero, exceeded 140 µm.

Dimensional analysis confirmed TP1 outperformed TP0 in terms of dimensional precision across most parameters.

**Figure 6:**
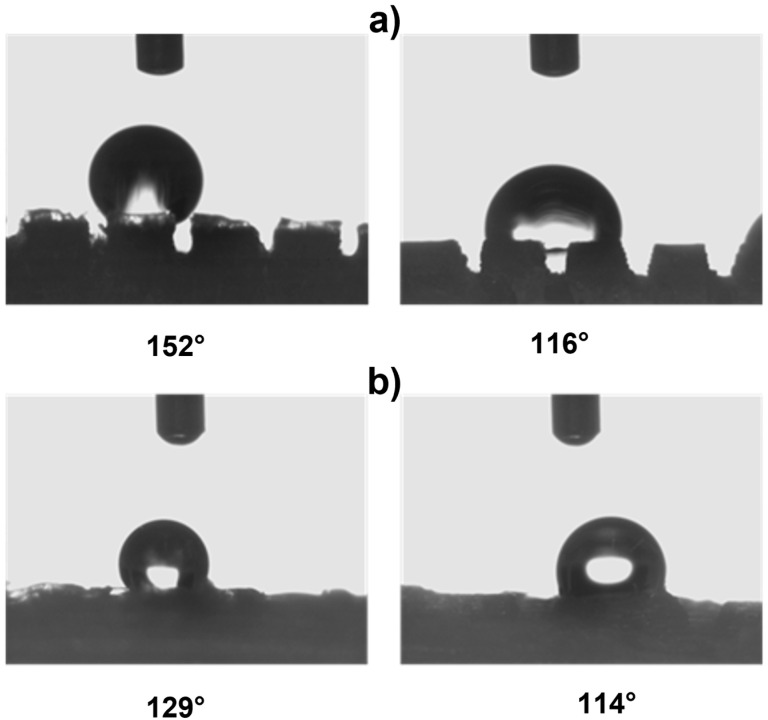
Wettability measurements: (**a**) on the pillar along the y-direction (left) and between the pillars (right) of TP0 sample; (**b**) along the y-direction (left) and the x-direction (right) of TP1 sample.

The wettability analysis of the TP0 sample along the y-direction reveals two distinct behaviors, depending on whether the water droplet rests atop a truncated pyramid or in the gap between two adjacent pyramids (Figure 6a). In the first case, the contact angle reaches up to a value of 152°, indicating a highly hydrophobic surface. In the second case, the contact angle decreases to 116° yet still reflects the characteristic features of the Cassie–Baxter regime, similar to what was observed in the TG sample. Increasing the droplet volume (e.g., doubling or tripling it) does not eliminate the two distinct wetting behaviors observed along the y-direction (see Figure S1). Along the x-direction, no significant variation in contact angle is detected, as the surface lacks structuring in that orientation. On the other hand, wettability measurements of TP1 revealed different contact angle values along the two principal directions: 129° along the y-axis and 114° along the x-axis (Figure 6b). Compared to sample TP0, TP1 showed a decrease in more than 20° in the y-direction (from 152° to 129°), probably due to the staggered arrangement of the structures. Along the x-direction, the introduction of surface structuring yielded promising results, contributing to the observed hydrophobic behavior.

Although no other examples of truncated pyramidal structures are currently available in the literature, a comparison can be made with pyramidal PLA samples fabricated via FFF [17,19]. Among these, the present case demonstrates the best performance in terms of achieving smaller printed feature dimensions and lower wettability without the need for chemical surface treatments.

In addition to the truncated pyramid structures, elliptical truncated cone (truncated conical geometry with ellipsoidal bases) geometries, referred to as CET, were also designed and fabricated. These structures were intended both to evaluate the printer resolution when producing rounded features and to investigate the influence of this type of geometry on surface wettability.

Different combinations of dimensions were tested, with the CET structures exhibiting a wide range of values. Specifically, dimension A ranged from 1.3 to 0.8 mm, B from 0.8 to 0.3 mm, a from 1.1 to 0.3 mm, and b from 0.6 to 0.2 mm. The spacings, spx and spy, varied from 0.2 mm to 0 and from 0.4 mm to 0, respectively. Additionally, the structures were arranged in both staggered (S) and non-staggered (NS) configurations. The geometric and dimensional characteristics of a representative CET sample (CET9_NS_h400) are listed in Table 2, while the corresponding data for all other CET samples can be found in Table S3 of the Supplementary Materials.

**Figure 7:**
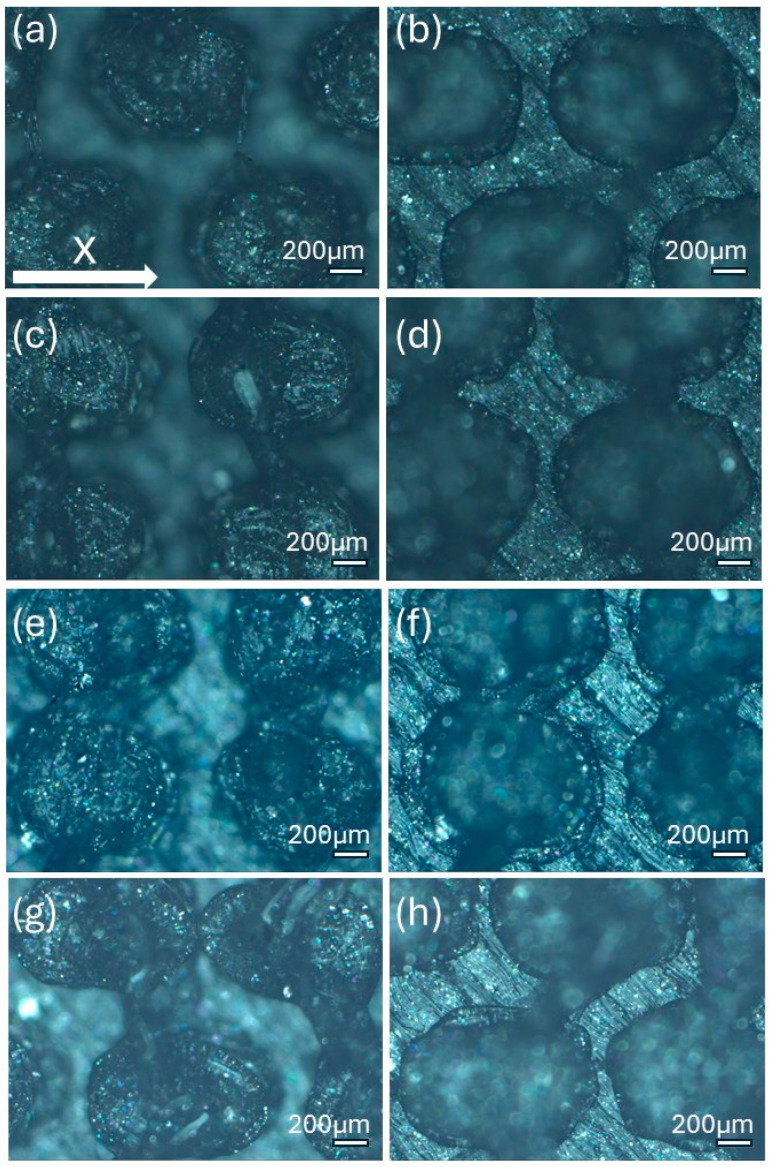
Optical microscopy images of CET9_S_h600 (**a**,**b**), CET9_NS_h600 (**c**,**d**), CET9_NS_h400 (**e**,**f**), and CET9_S_h400 (**g**,**h**). Each sample is shown from a top view with focus on the upper surface (**a**,**c**,**e**,**g**) and on the bottom surface (**b**,**d**,**f**,**h**).

The morphology of the samples was analyzed using optical microscopy. Figure 7 presents the images of the most optimized CET geometries, represented by the samples CET9_S_h600, CET9_NS_h600, CET9_NS_h400, and CET9_S_h400. These samples share identical dimensions for A′, B′, a′, and b′, but differ in height (h = 600 and 400 µm) and in the type of sequence—staggered (S) and non-staggered (NS).

The resulting pillars of all samples exhibit a truncated cone geometry with ellipsoidal bases, closely matching the CAD model used. A “stringing” effect between the pillars is more noticeable in the non-staggered structures (CET9_NS_h600, CET9_NS_h400) and in the staggered sample CET9_S_h400. However, it appears to be homogeneously distributed across the sample and presents a preferential orientation along the y-direction. No evident effect is observed in CET9_S_h600. In addition, the “stringing” effect does not affect the individual pillar geometry, which retains its geometrical identity. Through analysis of optical micrographs taken from different regions of the samples and processed using ZEN Core software, the average dimensions of the pillars and their spacing along the x and y axes were measured. The measured data are reported in Table 5.

Table 6 presents a comparison of the main fabricated geometries (TG, TP0, TP1, and CET9_NS_h400), highlighting differences between the CAD models (shown in black) and the printed geometries (shown in white), scaled at 25:1.

**Figure 8:**
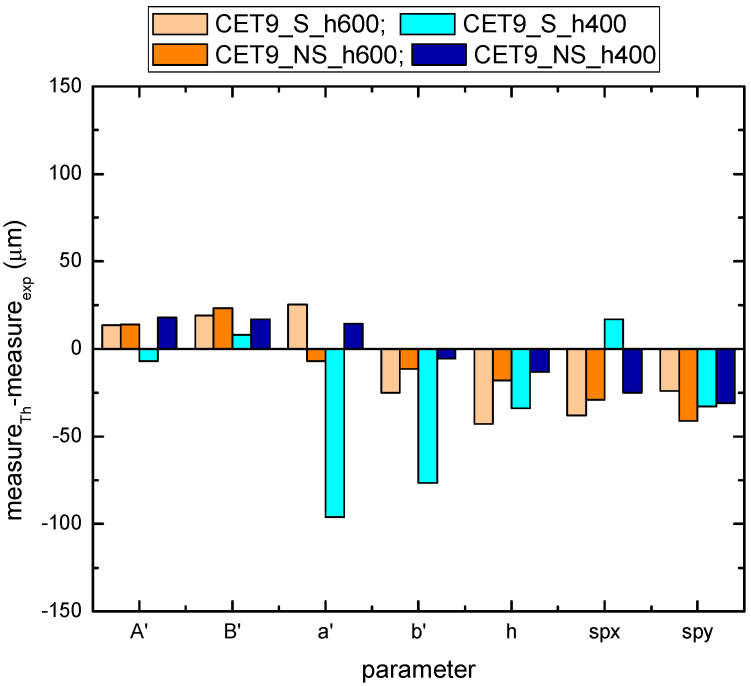
Deviations between theoretical (th) and experimental (exp) dimensions of CET structures.

For samples CET9_S_h600, CET9_NS_h600, and CET9_NS_h400, a good reproducibility of the pillar dimensions was observed, with values closely matching the theoretical specifications defined in the CAD designs. In contrast, sample CET9_S_h400 exhibited lower dimensional reproducibility, particularly in the measurements of dimensions a and b, representing the semi-major and semi-minor axes of the top base, respectively. Optical microscopy images (Figure 7) revealed that some of the pillars in CET9_S_h400 were partially fused together. This behavior contrasts with that of sample CET9_S_h600, which differs only in pillar height (400 µm for CET9_S_h400 vs. 600 µm for CET9_S_h600) while maintaining identical dimensions and the same staggered (S) structure. As matter of fact, CET9_S_h600 represents the best CET-printed geometry. As for TP samples, Figure 8 shows the dimensional deviations between theoretical and experimental values for four CET structure variants across seven parameters.

**Figure 9:**
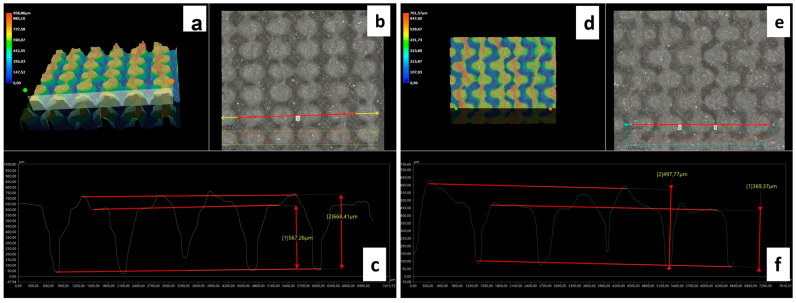
Optical and digital composition of 3D images (**a**,**d**); optical microscopy image of the top surface (**b**,**e**); profile analysis (**c**,**f**) of samples CET9_NS_h600 (left) and CET9_S_h400 (right) (scanned along the red line of (**b**,**e**), respectively).

The deviations slightly vary across both parameters and structure types. Overall, at both heights (600 and 400 µm), NS structures appear to align more closely with the theoretical dimensions compared to their S counterparts. For both S and NS structures, the configuration with h = 400 µm is preferred, as it shows better alignment with the theorical dimensions compared to the h = 600 µm variant. Figure 9 shows digital optical micrographs of samples CET9_NS_h600 (left) and CET9_S_h400 (right), captured using a Keyence VHX-X1 digital microscope. These images are representative of the CET geometry, illustrating the two investigated heights (h600 and h400), and the structural types (NS and S) designed for the study. The profile analysis confirmed the pillar height (h) dimensions in the samples investigated. Specifically, CET9_NS_h600 measured 618 µm and CET9_S_h400 showed a height of 434 µm. All the values of h are reported in Table 5. As with TP1, the h measurements were averaged over a small area, which may affect the accuracy of the results.

As with the TP geometry samples, wettability measurements were conducted on the CET geometry samples by evaluating the contact angle along both the x and y directions of each specimen. The most consistent and comparable water contact angle (WCA) values in both directions—along with a reduced tendency for the droplet to sink between the structures—were observed in samples with the following identical dimensions: A’ = 600 µm, B’ = 450 µm, a’ = 450 µm, b’ = 300 µm, and spacings spx and spy = 100 µm, namely CET9. These samples differed only in the height (h), which was 600 µm or 400 µm as well as in the sequence type: staggered (S) or not staggered (NS). This approach allows for the evaluation of how the height and position of microstructures affect the wettability of the samples. The corresponding WCA values are presented in Table 7. The WCA values of all CET samples fabricated are reported in Table S3 of Supplementary Materials.

Samples CET9_S_h600 and CET9_NS_h600, which differ solely in their structural arrangement—staggered (S) for CET9_S_h600 and non-staggered (NS) for CET9_NS_h600—exhibit markedly different water contact angles (WCA) along the x- and y-directions. This suggests that, in this case, the staggered configuration promotes a more hydrophobic surface, reducing the formation of great holes between the structure and favoring a wetting regime better described by the Cassie–Baxter model rather than the Wenzel model (see Figure 4). Furthermore, the absence of stringing increases hydrophobicity due to well-defined, isolated pillars.

Interestingly, sample CET9_NS_h400—identical to CET9_NS_h600 except for a reduced pillar height (400 µm instead of 600 µm)—exhibits a much higher WCA of about 128°, despite having a non-staggered structure. This behavior may be attributed to the reduced pillar height. As the structural height increases, the conical slope becomes steeper, potentially compromising the droplet ability to remain suspended in a Cassie–Baxter state and leading to increased liquid penetration. In contrast, this effect is not observable for CET9_S_h400—which features a staggered structure and the same reduced height as CET9_NS_h400—but shows significantly lower WCA values (80–90°) in both directions. Optical microscopy reveals that due to a pronouncing stringing effect, the pillars in CET9_S_h400 are partially fused, forming flat regions between them. These flat areas likely allow the droplet to spread more easily, diminishing the effect of the structured geometry.

Stringing effect was particularly evident in both CET9_NS_h600 and CET9_S_h400 where introduces defects and creates a surface roughness on 3D-printed samples which may hinder the formation of the air cushion necessary for water repellency or promote the adhesion of water droplets in undesired areas influencing hydrophobicity. Indeed, the WCA values approach those of the flat reference sample, indicating a diminished surface structuring effect.

**Figure 10:**
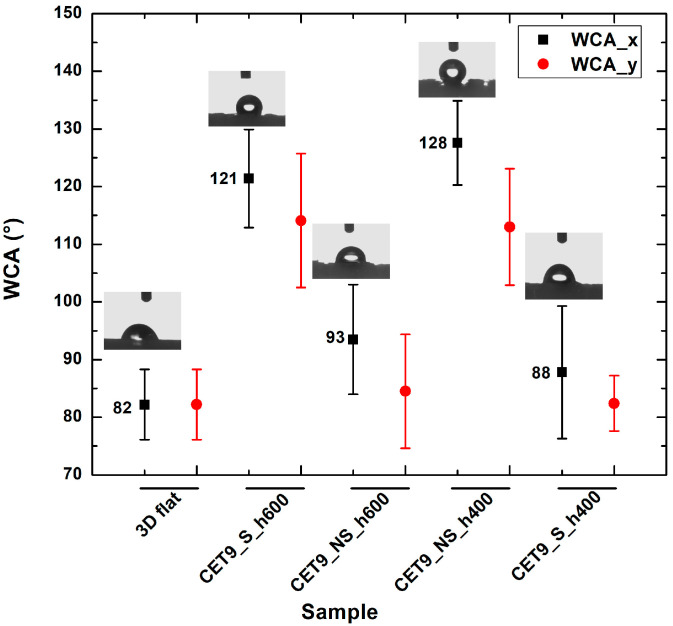
Water contact angles of samples CET9_S_h600, CET9_NS_h600, CET9_NS_h400, and CET9_S_h400 along the x-direction (black) and y-direction (red), and comparison with the unstructured sample (3D flat).

Figure 10 shows the trend in WCA for the best-performing CET structures compared to a flat, unstructured 3D-printed surface along the x- and y-directions. The highest increase was observed for sample CET9_NS_h400 (along with the x-direction), with an enhancement of approximately 55% respect to 3D flat sample (WCA = 82.2°). The same trend was observed along the y-direction with an improvement of approximately 37%.

Also in this case, the truncated conical geometry exhibits more hydrophobic behavior than the cylindrical and full conical geometry. Indeed, when comparing truncated ellipsoidal cone (CET) geometries with conventional cone structures, the CET design achieved WCA values of up to 127°, whereas cone geometries typically remained around 90° [19].

### 3.3. Chemical Treatment: Surface Deposition of SiO_2_ Nanoparticles

To evaluate a potential additional effect of surface treatments on the wettability of structured surfaces, a synthesis protocol was developed for SiO_2_ nanoparticles functionalized with a commercial fluoropolymer (Fluorolink S10) to be deposited on 3D samples. The hydrophobic properties of polymeric substrates (flat and patterned)—produced by fused filament fabrication (FFF) 3D printing—were then assessed after treatment with a hydrophobic coating (either the fluorinated polymer or the functionalized SiO_2_ nanoparticles).

#### 3.3.1. Preparation, Functionalization and Characterization of SiO_2_ Nanoparticles

**Figure 11:**
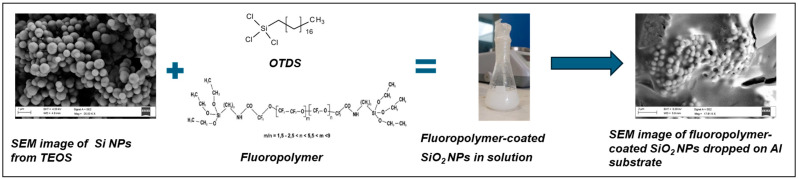
Schematic representation of the synthesis of SiO_2_ nanoparticles functionalized with Fluorolink S10.

Starting from tetraethyl orthosilicate (TEOS), silica nanoparticles were synthesized and subsequently functionalized with trichloro(octadecyl)silane (OTDS) and the commercial fluoropolymer Fluorolink S10. These reagents bind to the Si–O–Si groups, forming functionalized silica nanoparticles (SiO_2__S10). Fluorolink S10 contains two terminal silane groups and a high content of fluorocarbon chains, which impart the desired hydrophobic properties [20]. The synthesis process of nanoparticles is schematized in Figure 11.

**Figure 12:**
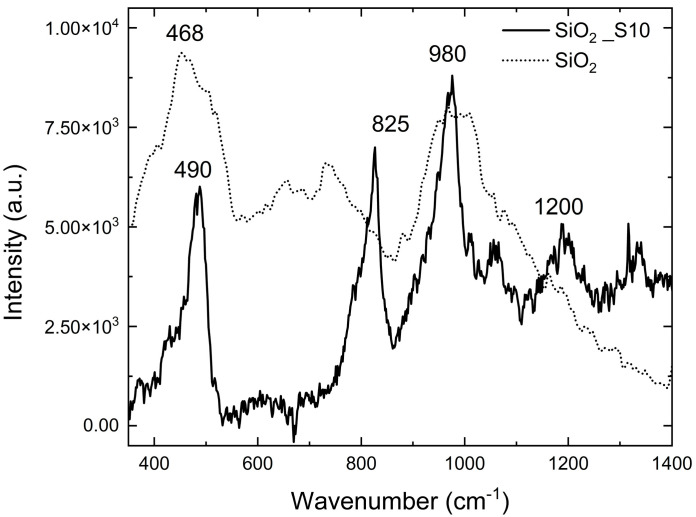
Raman spectra of SiO_2_ nanoparticles before (SiO_2_) and after functionalization with Fluorolink S10 (SiO_2__S10).

The nanoparticles were characterized using scanning electron microscopy (SEM), as shown in Figure 11, and Raman spectroscopy (Figure 12). SEM analysis revealed the formation of nanoparticles with an average diameter of approximately 400 nm, coated with polymer after functionalization. Figure 12 presents the Raman spectra (acquired using a 514 nm laser source) of the SiO_2_ nanoparticles before and after functionalization with the perfluoropolyether Fluorolink S10.

In the RAMAN spectrum of the unmodified silica nanoparticles (dot line), two prominent bands are observed, centered at 468 cm^−1^ and 980 cm^−1^. The first band, resulting from the overlap of multiple characteristic SiO_2_ signals, suggests that the material is present in nanoparticle morphology. The second band provides evidence of Si–OH bonds presence [25,26]. In the samples subjected to the functionalization process, several additional bands appear (continuous line). Among these, the band at 825 cm^−1^ can be attributed to the collective vibrational modes of C–O–C groups and the stretching vibrations of CF groups in the perfluoropolyether structure [27].

#### 3.3.2. Hydrophobic Coating Deposition

**Figure 13:**
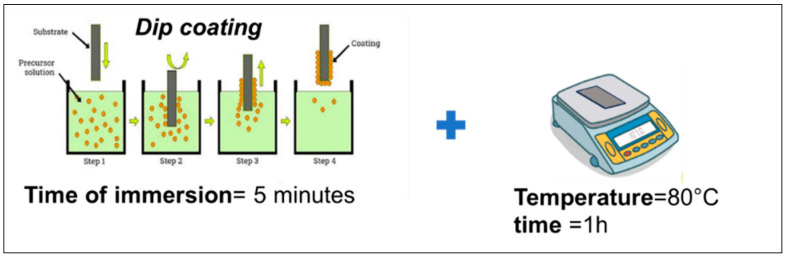
Schematic of the dip-coating process for hydrophobic layer deposition.

As the first attempts to further enhance the hydrophobic character, selected samples showing higher WCA values (TP0, TP1, and CET9_NS_h400) were subjected to surface functionalization through the application of a hydrophobic coating, using either a fluorinated polymer or functionalized SiO_2_ nanoparticles. Particularly, the substrates were immersed in isopropanol-based solution of Fluorolink S10 or SiO_2__S10 using a dip-coating process (see schematic in Figure 13). Each sample was kept in solution for 5 min, then slowly withdrawn, air-dried at room temperature, and subsequently heated on a hot plate at 80 °C for 1 h.

**Figure 14:**
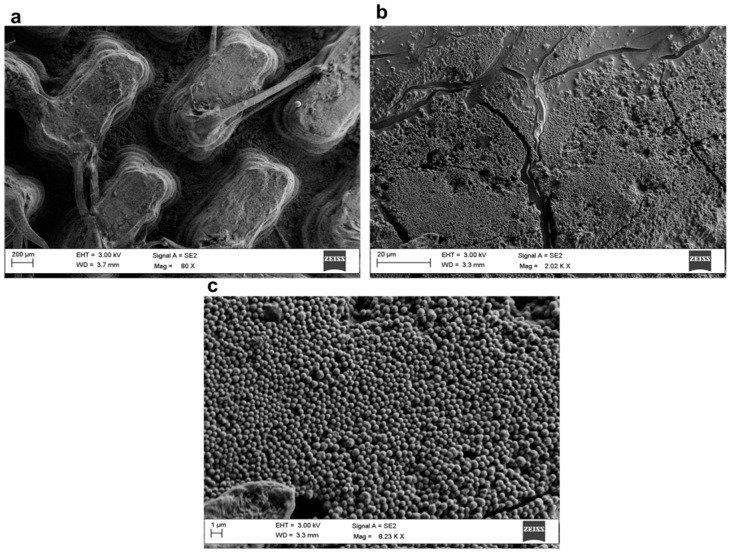
SEM images of the TP1-SiO_2__S10 coated sample at three different magnifications: 80× (**a**), 2000× (**b**), and 8200× (**c**).

**Figure 15:**
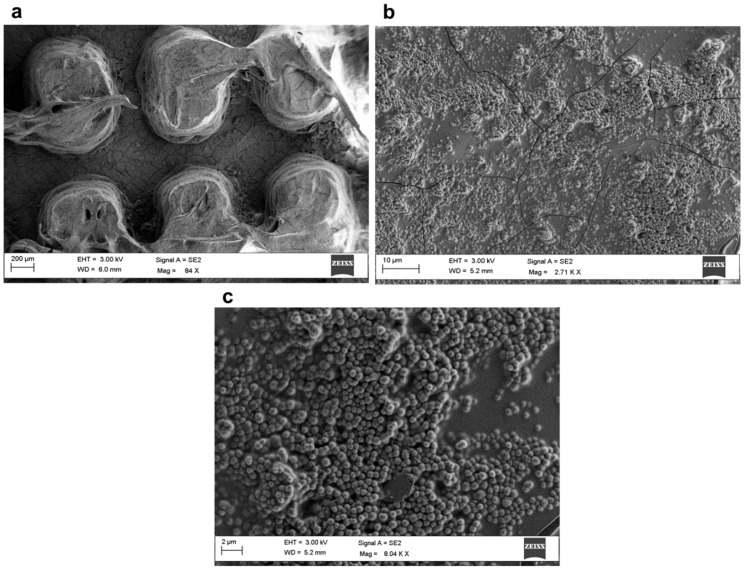
SEM images of the CET9_NS_h400-SiO_2__S10 coated sample at three different magnifications: 80× (**a**), 2700× (**b**), 8000× (**c**).

Figure 14 and Figure 15 show SEM images at three different magnifications of samples TP1-SiO_2__S10 coated and CET9_NS_h400-SiO_2__S10 coated, respectively. The high-magnification images (Figure 14c and Figure 15c) clearly reveal the spherical morphology of the nanoparticles deposited on the top surface of the 3D structures. The diameter of the nanoparticles range between 450 and 550 nm.

As shown in Figure 14b and Figure 15b, the deposited layer does not uniformly cover the entire surface; visible cracks and uncovered regions are present. This indicates that the nanoparticle deposition process requires further optimization to enhance both the continuity of the layer and its adhesion to the PLA substrate.

**Figure 16:**
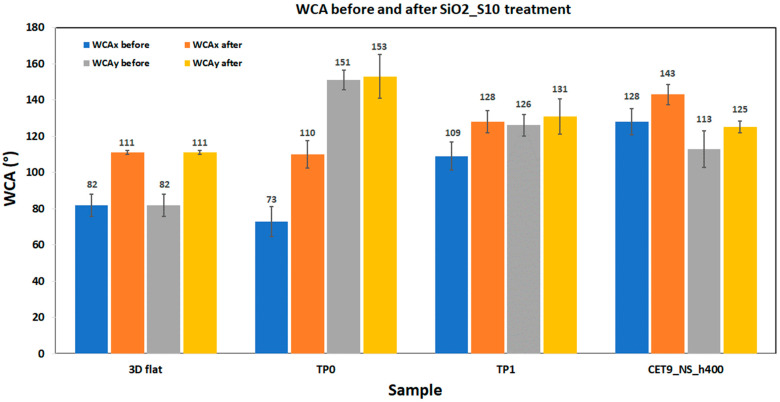
Comparative WCAx and WCAy values for 3D flat, TP0, TP1, and CET9_NS_h400 before and after SiO2_S10 treatment.

Table S4 in the Supplementary Materials reports the WCA values for the structured samples TP0, TP1, and CET9_NS_h400 after coating with SiO_2__S10, compared to the same samples before hydrophobic treatment. The table also includes the unstructured 3D flat sample as a reference. A graphical comparison of WCA values before and after SiO2_S10 treatment is reported in Figure 16. For the 3D flat sample, the hydrophobic coating enhances the WCA of the substrate of about 35%. Regarding the structured samples, TP0, which features structuring only along the y-axis, the coating with nanoparticles resulted in a negligible increase in WCA along the structured y-direction, while a significant increase was observed along the unstructured x-direction (about 49%). For the TP1 geometry, an additional increase was observed due to the SiO_2__S10 nanoparticle layer (18% in the x-direction and 3% in the y-direction), while for sample CET9_NS_h400 an additional increase of about 12% in the x-direction was registered, reaching a value of 143.5° after the application of the SiO_2__S10 coating.

Based on the results obtained, it is evident that under the conditions analyzed, 3D microstructuring via 3D-printing technique has proven to be more efficient and cost-effective in enhancing the material’s hydrophobicity than the initial attempts at depositing the synthesized nanometric coating layer. However, a more detailed optimization of the coating process—such as improving nanoparticle dispersion or enhancing the compatibility between the coating and the substrate—could potentially increase the effectiveness of the deposition and justify its added cost and complexity.

## 4. Conclusions

The ability to tailor surface properties through 3D printing and surface engineering allows the development of sustainable, high-performance polymer components. In this work, commercial PLA filament was used to fabricate three different 3D geometries by fused filament fabrication (FFF) technique. In particular, one pointed structure (TG, triangular-based prism) and two flat-based structures (TP, truncated pyramid, and CET, truncated ellipsoidal cone) were designed and printed. The study focused on exploring how variations in design strategies and the deposition of a fluoropolymer–SiO_2_ nanoparticle coating contribute to the hydrophobic behavior of the printed samples. All 3D-patterned samples showed a remarkable improvement in the hydrophobic behavior compared to the flat surface. The FFF technique shows slight deviations in shape and dimensions in pointed structure, as resulted in TG pattern. Nevertheless, the TG sample demonstrated superhydrophobic behavior along the structured y-direction, with a water contact angle (WCA) of approximately 164°. Likewise, the TP0 sample, also structured only along the y-direction, presented a WCA value exceeding 150°. In this case, a negligible effect of the coating on the surface wettability was detected. On the other hand, CET geometry showed the highest contact angle in the x-direction (127.6° for sample CET9_NS_h400), which increased by 12.5% after applying the hydrophobic coating, reaching a WCA of approximately 143°.

These findings suggest that the variations applied to commonly investigated microstructures enable, to the best of our knowledge, the fabrication of the smallest features using the FFF technique, while maintaining good resolution and achieving higher contact angles.

Furthermore, under the investigated conditions, the enhanced hydrophobic properties of the 3D-printed geometries appear to be primarily driven by microstructuring than by silica nanoparticle-based coating.

Future experimental designs should consider both the morphology of the surface to be treated (e.g., patterned structures) and the compatibility between the substrate and the nanoparticle coating.

## Figures and Tables

**Table 1:** Main printing parameters for all geometries fabricated.

Filament	Material	Nozzle Diameter (mm)	Nozzle Temperature (°C)	Bed Temperature (°C)	Infill Type	Printing Speed (mm/s)	Layer Thickness (mm)	Infill Density (%)
Galaxy silver Prusa	PLA	0.2	215	60	linear	25	0.2 first layer 0.05 other layers	100

**Table 2:** Details of the FFF 3D-printed samples: flat surface (F); triangular-based prism (TG); truncated pyramid (TP); truncated ellipsoidal cone (CET). CAD design: top view (1); 3D view (2); lateral view (3). Dimensions of TP and CET refer to selected specimens.

Sample	Surface Morphology	CAD Design	Dimension (μm)
F	Without patterns	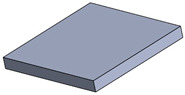	Flat surface
TG	Triangular prism	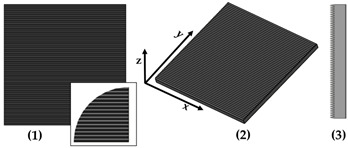	L: specimens’ length B: 400 h: 400 spy: 0 NS
TP0	Truncated pyramid	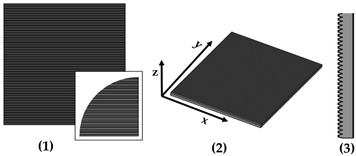	a: 800 b: specimens’ length c: 400 d: specimens’ length h: 400 spy: 0 NS
TP1	Truncated pyramid	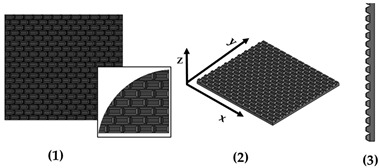	a: 800 b: 1500 c: 400 d: 1100 h: 400 spx, spy: 100 S
CET9_NS_h400	Truncated cone	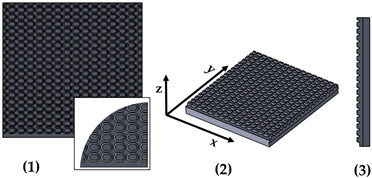	A′: 600 B′: 450 a′: 450 b′: 300 h: 400 spx, spy: 100 NS
**Legend**			
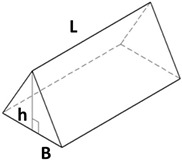		TG: Triangular prism L: length of the prism B: bottom edge of the base triangle h: height of the base triangle	
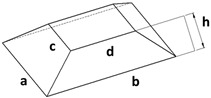		TP: Truncated pyramid a: shorter side of the lower base b: longer side of the lower base c: shorter side of the upper base d: longer side of the upper base h: truncated height	
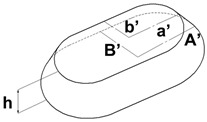		CET: Truncated ellipsoidal cone A′: semi-major axis of the bottom base B′: semi-minor axis of the bottom base a′: semi-major axis of the top base b′: semi-minor axis of the top base h: truncated height	
spx: spacing between patterns along x-axis spy: spacing between patterns along y-axis S: sequence of staggered structures NS: sequence of non-staggered structures			

**Table 3:** Theoretical (th, from CAD design) and experimental (exp) dimensions of TG structure measured with Zen Core software on optical microscopy images. a: lower base (short side); c: upper base (short side); h: height; spy: spacing between patterns along y-axis. Image comparing the CAD model (black) and the print geometry (white).

Sample		a (µm)	c (µm)	h (µm)	Spy (µm)	CAD Model (Black) Print Geometry (White) Scale 25:1	Axis View
TG	th	400	0	400	0	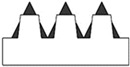	y
	exp	262 ± 16	202 ± 11	255 ± 4	134 ± 15		

**Table 4:** Theoretical (th, from CAD design) and experimental (exp) dimensions of TP structures measured with Zen Core software on optical microscopy images. a and b: shorter and longer side of the lower base, respectively; c and d: shorter and longer side of the upper base, respectively; h: vertical distance between the upper and lower base; spx and spy: spacing between patterns along x-axis and y-axis, respectively. sp length means the length of the specimen. Image comparing the CAD model (black) and the print geometry (white).

Sample		a (µm)	b (µm)	c (µm)	d (µm)	h (µm)	spx (µm)	spy (µm)	CAD Model (Black) Print Geometry (White). Scale 10:1	Axis View
TP0	th	800	sp length	400	sp length	400	/	0	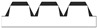	y
	exp	618 ± 54	sp length	520 ± 27	sp length	302 ± 3	/	140 ± 17		
TP1	th	800	1500	400	1100	400	100	100	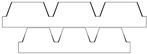	x y
	exp	724 ± 22	1436 ± 21	432 ± 29	1034 ± 85	325 ± 42	151 ± 20	153 ± 14		

**Table 5:** Theoretical (th, from CAD design) and experimental (exp) dimensions of CET structures measured with Zen Core software on optical microscopy images; 2 *A′ and 2 *B′: major and minor axis of the bottom base, respectively; 2 *a′ and 2 *b′: major and minor axis of the top base, respectively; h: truncated height; spx and spy: spacing between patterns along x-axis and y-axis, respectively. Image comparing the CAD model (black) and the print geometry (white).

Sample		2 *A′ (µm)	2 *B′ (µm)	2 *a′ (µm)	2 *b’ (µm)	h (µm)	spx (µm)	spy (µm)	CAD Model (Black) Print Geometry (White) Scale 10:1	Axis View
CET9_S_h600	th	1200	900	900	600	600	100	100	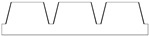	x
	exp	1173 ± 26	862 ± 22	849 ± 51	650 ± 46	643 ± 57	138 ± 26	124 ± 30	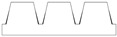	y
CET9_NS_h600	th	1200	900	900	600	600	100	100	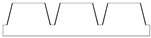	x
	exp	1172 ± 34	853 ± 30	914 ± 81	623 ± 47	618 ± 50	129 ± 32	141 ± 14	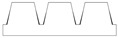	y
CET9_NS_h400	th	1200	900	900	600	400	100	100	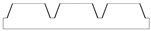	x
	exp	1164 ± 39	866 ± 16	871 ± 30	611 ± 40	413 ± 34	125 ± 23	131 ± 20	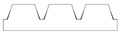	y
CET9_S_h400	th	1200	900	900	600	400	100	100	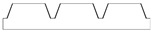	x
	exp	1214 ± 62	884 ± 33	1092 ± 48	753 ± 52	434 ± 64	83 ± 34	133 ± 32	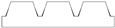	y

**Table 6:** Comparison of the CAD model (black) and the print geometry (white).

Sample	CAD Model (Black) vs. Print Geometry (White) Scale 25:1	Axis View
TG	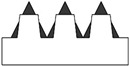	x
TP0	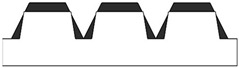	x
TP1	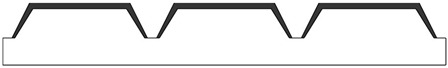	x
	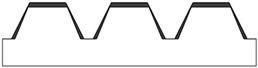	y
CET9_NS_h400	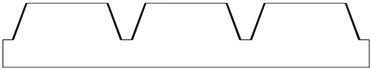	x
	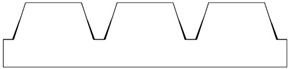	y

**Table 7:** Water contact angle values for the samples with the best optimized CET geometries evaluated along the x-direction (WCAx) and the y-direction (WCAy) of each specimen.

Sample	WCAx (°)	WCAy (°)
CET9_S_h600	121.4 ± 8.5	114.1 ± 11.6
CET9_NS_h600	93.5 ± 9.5	84.5 ± 9.9
CET9_NS_h400	127.6 ± 7.3	113.0 ± 10.1
CET9_S_h400	87.8 ± 11.5	82.4 ± 4.8

## Data Availability

The original contributions presented in this study are included in the article/Supplementary Material. Further inquiries can be directed to the corresponding author.

## Supplementary Materials

The following supporting information can be downloaded at https://www.mdpi.com/article/10.3390/polym17192570/s1, Table S1: Details of the FFF 3D printed samples with TG geometry: CAD dimension and water contact angle; Table S2: Details of the FFF 3D printed samples with TP geometry: CAD dimension and water contact angle; Table S3: Details of the FFF 3D printed samples with CET geometry. Dimension, water contact angle and photo of the specimens; Table S4: Water contact angle values for samples 3D flat, TP0, TP1, CET9_NS_h400 before and after SiO2_S10 deposition. Figure S1: Wettability images of the TP0 sample. Top: on the pillar along the y-direction (left), and with doubled drop volume (right). Bottom: between the pillars along the y-direction (left), and with doubled drop volume (right).

## Abbreviations

The following abbreviations are used in this manuscript:

PLA	Polylactic Acid
FFF	Fused Filament Fabrication
WCA	Water Contact Angle
TG	Triangular-based Prism
TP	Truncated Pyramid
CET	Truncated Ellipsoidal Cone
SEM	Scanning Electron Microscopy
TEOS	Tetraethyl Orthosilicate
OTDS	Trichloro(octadecyl)silane
NPs	Nanoparticles
