# Supplementary material for: Three-Dimensionally Printed Microstructured Hydrophobic Surfaces: Morphology and Wettability
Source: Polymers (Basel). 2025 Sep 23;17(19):2570. doi: 10.3390/polym17192570 (PMC12526174; doi:10.3390/polym17192570)
Supplement: Supplementary file 1 [file polymers-17-02570-s001.zip › polymers-3825336-supplementary.pdf]

Table S1. Details of the FFF 3D printed samples with TG geometry: CAD dimension and water contact angle.

**GEOMETRY**	**LEGEND**	
**Triangular prism (TG)**<br>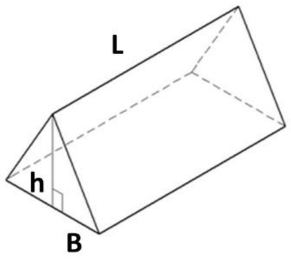 	L: length of the prism<br>B: bottom edge of the base triangle<br>h: height of the base triangle<br>spy: spacing between patterns along y-axis<br>NS: sequence of non-staggered structures<br>WCAx: Water contact angle evaluated along the x-direction<br>WCAy: Water contact angle evaluated along the y-direction	
**Sample name**	**CAD dimension (µm)**	**Water Contact Angle (°)**
**TG**	L: specimens' length<br>B: 400<br>h: 400<br>spy: 0<br>NS	WCAx: 164 ± 8.6<br>WCAy: 74.9 ± 5.5

Table S2. Details of the FFF 3D printed samples with TP geometry: CAD dimension and water contact angle.

**GEOMETRY**	**LEGEND**	
**Truncated pyramid (TP)**<br>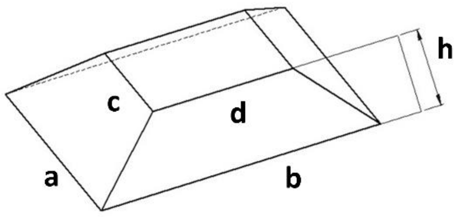 	a: shorter side of the lower base<br>b: longer side of the lower base<br>c: shorter side of the upper base<br>d: longer side of the upper base<br>h: truncated height<br>spx: spacing between patterns along x-axis<br>spy: spacing between patterns along y-axis<br>NS: sequence of non-staggered structures<br>S: sequence of staggered structures<br>WCAx: Water contact angle evaluated along the x-direction<br>WCAy: Water contact angle evaluated along the y-direction	
**Sample name**	**CAD dimension**<br>**(μm)**	**Water Contact Angle**<br>**(°)**
**TP0**	a: 800<br>b: specimen length<br>c: 400<br>d: specimen length<br>h: 400<br>spy: 0<br>NS	WCAx: 73.4 ± 8.3<br>WCAy: 151.4 ± 5.4
**TP1**	a: 800<br>b: 1500<br>c: 400<br>d: 1100<br>h: 400<br>spx: 100<br>spy: 100<br>S	WCAx: 108.7 ± 7.7<br>WCAy: 126.3 ± 6.1
**TP1_NS**	a: 800<br>b: 1500	WCAx 111.0 ± 3.9

	c: 400<br>d: 1100<br>h: 400<br>spx: 100<br>spy: 100<br>NS	WCAy :105.9 ± 12.1

Table S3. Details of the FFF 3D printed samples with CET geometry. Dimension, water contact angle and photo of the specimens.

**GEOMETRY**	**LEGEND**		
**Truncated ellipsoidal cone (CET)**<br>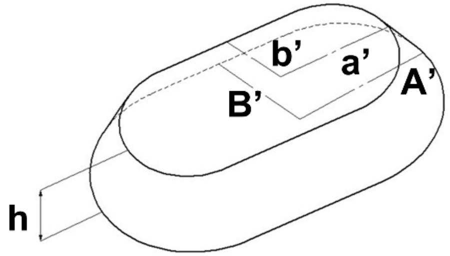	A′: semi-major axis of the bottom base<br>B′: semi-minor axis of the bottom base<br>a': semi-major axis of the top base<br>b′: semi-minor axis of the top base<br>h: truncated height<br>spx: spacing between patterns along x-axis<br>spy: spacing between patterns along y-axis<br>NS: sequence of non-staggered structures<br>S: sequence of staggered structures<br>WCAx: Water contact angle evaluated along the x-direction<br>WCAy: Water contact angle evaluated along the y-direction		
**Sample name**	**CAD dimension (µm)**	**Water Contact Angle (°)**	**Photo of the 3D specimen**
**CET0**	A′: 500<br>B′: 500<br>a′: 300<br>b′: 300<br>h: 600<br>spx:0<br>spy: 0<br>NS	WCAx, WCAy: 82.4 ± 6.6	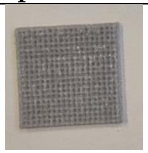
**CET1**	A′: 1300<br>B′: 800<br>a′: 1100<br>b′: 600<br>h: 600<br>spx: 200<br>spy: 400<br>S	WCAx: 89.7 ± 9.7<br>WCAy: 114.1 ± 7.4	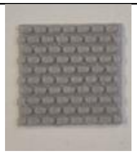

**CET2**	A′: 1300<br>B′: 800<br>a′:1100<br>b′:600<br>h: 600<br>spx: 200<br>spy: 200<br>S	WCAx: 84.8 ± 4.9<br>WCAy: 117.5 ± 11.3	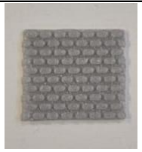
**CET3**	A′: 1100<br>B′: 600<br>a′: 900<br>b′: 400<br>h: 600<br>spx: 100<br>spy: 100<br>S	WCAx: 101.6 ± 6.0<br>WCAy: 123.5 ± 10.0	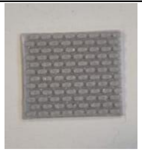
**CET4**	A′: 1100<br>B′: 600<br>b′: 900<br>b′: 400<br>h: 600<br>spx: 200<br>spy: 200<br>S	WCAx: 90.7 ± 3.5<br>WCAy: 131.6 ± 1.8	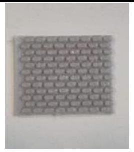
**CET5**	A′: 800<br>B′: 600<br>a′: 600<br>b′: 400<br>h: 600<br>spx: 100<br>spy: 100<br>S	WCAx: 106.3 ± 4.2<br>WCAy: 130.5 ± 5.4	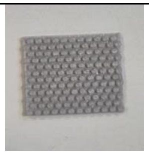

**CET6**	A′: 800<br>B′: 600<br>a′: 600<br>b′: 400<br>h: 600<br>spx: 0<br>spy: 0<br>S	WCAx: 118.3 ± 4.2<br>WCAy: 137.0 ± 4.8	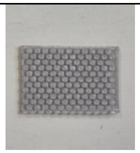
**CET7_S**	A′: 400<br>B′: 300<br>a′: 300<br>b′: 200<br>h: 600<br>spx: 100<br>spy: 100<br>S	WCAx: 94.7 ± 5.1<br>WCAy: 125.7 ± 14.5	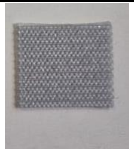
**CET7_NS**	A′: 400<br>B′: 300<br>a′: 300<br>b′: 200<br>h: 600<br>spx: 100<br>spy: 100<br>NS	WCAx: 95.9 ±2.1<br>WCAy: 94.3 ± 11.3	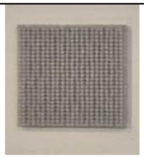
**CET8**	A′: 400<br>B′: 300<br>a′: 300<br>b′: 200<br>h: 600<br>spx: 100-200 (alternating values)<br>spy: 100<br>S	WCAx: 99.0 ± 5.5<br>WCAy: 127.5 ± 12.3	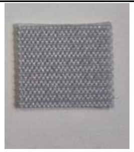

**CET9_S_h600**	A′: 600<br>B′: 450<br>a′: 450<br>b′: 300<br>h: 600<br>spx: 100<br>spy: 100<br>S	WCAx: 121.4 ± 8.5<br>WCAy: 114.1 ± 11.6	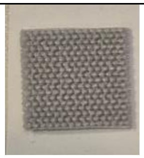
**CET9_NS_h600**	A′: 600<br>B′: 450<br>a′: 450<br>b′: 300<br>h: 600<br>spx: 100<br>spy: 100<br>NS	WCAx: 84.5 ± 9.9<br>WCAy: 93.5 ± 9.5	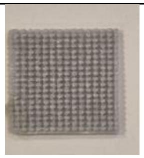
**CET9_NS_h400**	A′: 600<br>B′: 450<br>a′: 450<br>b′: 300<br>h: 400<br>spx: 100<br>spy: 100<br>NS	WCAx: 127.6 ± 7.3<br>WCAy: 113.0 ±10.1	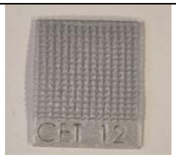
**CET9_S_h400**	A′: 600<br>B′: 450<br>a′: 450<br>b′: 300<br>h: 400<br>spx: 100<br>spy: 100<br>S	WCAx: 87.8 ± 11.5<br>WCAy: 82.4 ± 4.8	

Table S4. Water contact angle values for samples 3D flat, TP0, TP1, CET9_NS_h400 before and after SiO2_S10 deposition.

Sample	WCAx before (°)	WCAx after (°)	WCAy before (°)	WCAy after (°)
3D flat	82.2 ± 6.1	110.8 ± 1.1	82.2 ± 6.1	110.8 ± 1.1
TP0	73.4 ± 8.3	109.7 ± 7.7	151.4 ± 5.4	152.6 ± 6.9
TP1	108.7 ± 7.7	128.0 ± 6.2	126.3 ± 6.1	130.6 ± 9.7
CET9_NS_h400	127.6 ± 7.3	143.5 ± 5.6	113.0±10.1	125.2 ± 3.3

Figure S1. Wettability images of the TP0 sample. Top: on the pillar along the y-direction (left), and with doubled drop volume (right). Bottom: between the pillars along the y-direction (left), and with doubled drop volume (right).

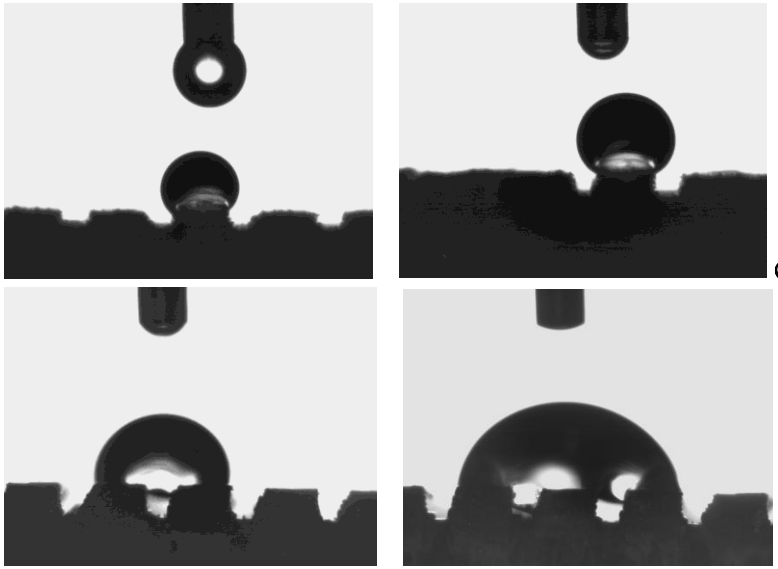

On top

Between adjacent structures

As shown in the figures below, increasing the droplet volume (e.g., doubling or tripling it) does not eliminate the two distinct wetting behaviors observed along the y-direction—namely, on top of or between adjacent structures. However, it is plausible that a further significant increase in droplet volume could lead to a substantial enlargement of the droplet footprint. This might cause the droplet to span across multiple adjacent pillars, potentially reducing or even eliminating the distinction between the two behaviors due to increased lateral spreading.
